# Neuro-ophthalmologic outcomes of standard versus hypo-fractionated stereotactic radiotherapy of AVPM

**DOI:** 10.1186/s13014-021-01879-2

**Published:** 2021-08-28

**Authors:** Leor Zach, Amir Agami, Orit Furman, Moshe Attia, Zvi Cohen, Iris Ben-Bassat Mizrachi, Guy Tam, Zion Zibly, Ouzi Nissim, Roberto Spiegelmann, Ruth Huna-Baron

**Affiliations:** 1 Neuro-Oncology Unit, Sheba Medical Center, Ramat Gan, Israel; 2 Neuro-Ophthalmology Service, Sheba Medical Center, Ramat Gan, Israel; 3 Neurosurgery Department, Sheba Medical Center, Ramat Gan, Israel; 4 Sackler Faculty of Medicine, Tel Aviv University, Tel Aviv, Israel; 5 Present Address: Galilee Medical Center, Nahariyya, Israel

## Abstract

**Background:**

Most anterior visual pathway meningiomas (AVPM) are benign and slow-growing, but these tumors may affect visual functions, including visual acuity (VA) and visual field (VF). Due to location, most are treated non-surgically by fractionated stereotactic radiotherapy (FSRT), aiming to prevent tumor progression and visual functions deterioration. Unfortunately, FSRT in itself may affect visual functions. The current preferred treatment regimen (in terms of safety and effectiveness) is undetermined. While most cases are treated with conventional fractionation (cFSRT)—50.4–54 Gy in 28–30 fractions of 1.8–2 Gy, advances in technology have allowed shortening of total treatment length to hypofractionation (hSRT)—25-27 Gy in 3–5 fractions of 5–9 Gy. Our aim was to evaluate the association of radiotherapy regimen for treating AVPM (cFSRT vs. hSRT) with visual function outcomes (VA, VF) at the last neuro-ophthalmologic evaluation.

**Methods:**

We conducted a retrospective cohort study of AVPM cases treated at Sheba Medical Center during 2004–2015. We compared cFSRT and hSRT regimens regarding visual function (VA, VF) outcomes at the last neuro-ophthalmologic evaluation. VA was determined by the logarithm of the minimum angle of resolution (LogMAR). VF was determined by the mean deviation (MD). A clinically relevant change in VA was defined as 0.2 LogMAR.

**Results:**

48 patients (13 receiving hSRT, 35 receiving cFSRT) were included, with a median follow-up of 55 months. No significant difference was evident regarding LogMAR or MD of involved eyes at the last evaluation. Six (17%) patients in the cFSRT group experienced clinically relevant VA deterioration in the involved eye, compared with six (46%) in hSRT (*p* = 0.06).

**Conclusion:**

Our findings, using comprehensive and meticulous investigation of visual outcomes, suggest that hSRT may be associated with higher risk for VA and VF deterioration in AVPM especially in ONSM. We recommend the use of cFSRT for ONSM.

**Supplementary Information:**

The online version contains supplementary material available at 10.1186/s13014-021-01879-2.

## Introduction

Anterior visual pathway meningioma (AVPM) in most cases are benign, slow growing WHO grade I tumors but may impair one or more visual functions: visual acuity (VA), visual field (VF), or color vision [1]. The tumor may cause diplopia due to cranial nerves III, IV and VI or orbital involvement [1, 2]. Without treatment, deterioration of vision functions [3] and finally blindness [4] occur.

In most cases, AVPM are diagnosed by imaging (without pathological verification) and are not operable. The common treatment is FSRT [5], a highly accurate radiation therapy, producing a patient/tumor-specific 3D coordinate system used throughout the treatment [6]. The treatment aim is to stop tumor growth and prevent deterioration of visual functions. The optimal fractionation scheme—the daily dose of radiation and the total number of doses—for safe and effective treatment of AVPM is still undetermined [7–11]. AVP structures exhibit greater sensitivity to single-fraction irradiation than other cranial nerves [8, 12]. In many AVPM cases, there is an impairment in visual functions even before treatment is given [3]. Radiation toxicity can damage optic pathways and impair their functions, particularly by radiation-induced optic neuropathy (RION), most likely by ischemic damage followed by necrosis, typically after 1 to 1.5 years [13]. Important risk factors for RION include total radiation dose (over 50 Gy), higher dose per radiation fraction, advanced age, previous exposure to chemotherapy, or optic nerve compromise at the beginning of radiation therapy [13]. Worse prognosis is noted with larger tumor volumes and higher tumor grades [14, 15].

Rogers et al. reviewed the treatment of AVPM and concluded that a total dose of up to 50 Gy for optic nerve sheath meningioma (ONSM) and AVPM produced good results [14]. Good results were also published by Stiebel-Kalish et al. using cFSRT (1.7-2 Gy / fraction, for a total of 50-60 Gy) for AVPM [5] with almost 90% control rate in a mean follow-up of 39mo. Visual function improved or stabilized in 8 of the 16 patients and worsened in 2 (12%). In published data, we found permanent deterioration in visual acuity or visual field in only 23 out of 1191 patients after cFSRT (2.1%, see Additional File 1 for references). This rate corresponds to the risk of damage to optic nerves and chiasm summarized by Mayo et al. [16], based mostly on descriptive publications with relatively small samples. The differences between published studies in outcome variables, therapeutic equipment, and follow-up periods make it difficult to reach conclusions, especially since some studies did not consider tumor progression (PD) as a variable that may affect the outcome.

Radiobiological models suggest hypo-fractionated regimens are more effective, while shortening treatment courses (hSRT) [17]. Choosing between these regimens for meningiomas in direct contact with the AVP is a double-edged sword: high-dose fractions reaching the sensitive blood supply of the optical system could lead to vascular damage and late secondary toxicity with loss of vision; on the other hand, a suboptimal dose could cause visual function deterioration as a result of PD.

Several series of patients with AVPM treated by hSRT have been published, with the caveat of insufficient reporting of dosimetric analysis for AVP structures or detailed measurement of visual function [7, 18–21].

In our institution, most AVPM cases received conventionally fractionated stereotactic radiotherapy (cFSRT), employing 28–30 daily doses of 1.8–2 Gy per day for a total dose of up to 54 Gy. With the introduction of new hypo-fractionated regimens and better on board imaging (OBI) instruments we started to treat patients with hSRT to AVPMs and even ONSMs. In light of the paucity of sufficiently detailed information, the present study investigated the relationship between the radiation therapy regimen (hSRT vs. cFSRT) and the change in visual function (visual acuity and visual fields), measured at last neuro-ophthalmologic evaluation compared to pre-treatment.

## Materials and methods

We conducted a retrospective cohort study of patients with AVPM treated with hSRT or cFSRT at Sheba Medical Center during 2004–2015. After receiving the local IRB approval, the data were extracted from patients’ computerized records. Statistical analysis was performed on anonymized data.

### Patient population

Patients were entered into analysis according to the following inclusion criteria: (1) patient received a radiological diagnosis of meningioma, (2) the tumor involved or was in anatomical proximity to one or more of the following locations: medial sphenoid wing; cavernous sinus; orbital apex; optic nerve sheath; tuberculum sella, (3) the tumor was treated with radiotherapy using hSRT or cFSRT protocols at Sheba Medical Center during 2004–2015, (4) data on neuro-ophthalmology and neuroimaging were available. Exclusion criteria included: (1) lack of visual acuity documentation in the involved eye before treatment, (2) patient underwent additional radiation therapy or surgery in the period between the first radiotherapy treatment and the first neuro-ophthalmologic assessment, (3) history of prior treatment with stereotactic radiosurgery (SRS), or (4) patient with no light perception (NLP) in the involved eye before treatment.

For each patient, we collected demographic variables (age at the time of treatment, gender, smoking history, and duration from symptom onset to diagnosis), pathology report of WHO grade of the tumor (if available), presenting signs and symptoms (headaches, seizures, blurred vision, ptosis, whether the tumor was an incidental finding) and medical history (previous surgery for the same tumor, prior radiation exposure, diagnosis of neurofibromatosis, vision-threatening systemic conditions such as diabetes, hypertension, collagen vascular disorders, chronic eye disease) (Table 1).

**Table 1:** Baseline characteristics of AVPM patients by treatment group

Characteristic	Treatment group		*p*-value
	cFSRT (n = 35)	hSRT (n = 13)	
*MRI Findings before treatment*			
Optic nerve sheath involved (including ONSM)/Not involved	22/13 (63%/37%)	6/7 (46%/54%)	0.297
Multiple masses	6 (18%)	1 (8%)	0.655
Radiographic laterality of tumor (right side)	16 (46%)	6 (46%)	0.978
Histopathologic classification (WHO Grade)			
*WHO Grade*			
Unknown	9 (56%)	4 (40%)	0.840
I	6 (38%)	5 (50%)	
II	1 (6%)	1 (10%)	
III	0 (0%)	0 (0%)	

*AVPM* anterior visual pathway meningioma; *hSRT* hypofractionated stereotactic radiotherapy; *cFSRT* conventionally fractionated stereotactic radiotherapy; *inv.* involvement; *ONSM* optic nerve sheath meningioma. *WHO* world health organization

We also reviewed data from the neuro-ophthalmologic examination before and after the radiotherapy. Best corrected visual acuity was determined by a neuro-ophthalmologist using Snellen chart. Visual fields were examined by Humphrey automated static perimetry (HVF). The evaluation included optic disc abnormality (no/atrophy/swelling), other cranial nerve abnormalities (III, IV, V1, V2, V3, VI, VII), the diagnosis of RION, radiation retinopathy, and other radiotherapy complications.

### Variable definitions

Visual acuity (VA) is a continuous quantitative variable, determined according to the logarithm of the minimum angle of resolution (LogMAR) [22]. We used a value of 1/400 Snellen chart (LogMAR = 2.6) to represent counting fingers (CF) and estimated values of LogMAR 2.7, 2.8, 2.9 to represent hand movement (HM), light perception (LP), and no light perception (NLP), respectively [23]. We defined variables related to change over the follow-up period to counteract the variance in pretreatment visual function evaluation. The VA change between pre-treatment evaluation and last evaluation was defined as: ΔLogMAR = last LogMAR minus pre-treatment LogMAR. A difference of 0.2 LogMAR (two lines on a Snellen chart) was defined as a clinically relevant change in VA. In many studies examining visual outcome in cases of tumors affecting anterior visual pathways, a change of 0.1 LogMar is used as significant [24]. We decided to define VA change of 0.2 which represents change of 2 lines in the visual acuity measurement as significant, in order to increase the specificity of the results, as only patients showing unequivocal deterioration were entered into the analysis [25].

The visual field (VF) is a continuous quantitative variable determined according to the mean deviation (MD) value in the last assessment documented [22]. The VF change from the pre-treatment evaluation to the last evaluation was defined as ΔMD = last MD minus pre-treatment MD. Radiotherapy treatment was defined as hSRT if it involved five fractions and as cFSRT if it involved 25–30 fractions. In cases of AVPM with bilateral effect, the worse eye (right or left) was determined according to the most recent neuro-ophthalmological assessment before treatment or by the side involved in the last pre-irradiation imaging examination. Visual function results were analyzed separately for “worse” and “better” eyes.

For patients who underwent further treatment after the cFSRT/hSRT (surgery or additional cranial re-irradiation), visual acuity and visual fields were determined according to the most recent pre-treatment assessment.

### Statistical analysis

Categorical variables were described as numbers and percentages. Continuous variables were reported as the median and interquartile (25%-75%) range (IQR). Categorical variables were compared between the groups using the chi-square test or Fisher's exact test as appropriate. Continuous variables were compared between the groups using the Mann–Whitney U test. Changes in continuous variables—between baseline and last assessment—were evaluated using Wilcoxon's signed-rank test. All statistical tests were two-sided. *p*-value < 0.05 was considered statistically significant. Statistical analysis was performed using SPSS software (IBM SPSS Statistics for Windows, version 25, IBM Corp., Armonk, NY, USA, 2017).

### Imaging data

We documented tumor location (right/left/bilateral), orbital involvement, and tumor origin (optic nerve sheath, sphenoid wing, cavernous sinus, tuberculum sella (including parasellar/suprasellar), clinoid (either anterior or posterior)) from the brain and orbits Magnetic Resonance Imaging (MRI) reports before the treatment (Table 1).

### Radiation therapy data

All the patients were treated in Sheba Medical Center in Israel. IPlan software (Brainlab) was used for treatment planning for all patients, Novalis True Beam Linear Accelerator (Varian/Brain lab) was used to treat the patients. Computerized medical records of the patients were retrieved, and data were collected regarding the date of treatment, the number of fractions, the daily radiation dose, and the total radiation dose. The dose to all anterior optic structures was limited to 54 Gy (EQD2) in all regimens used. The radiotherapy plans were not available for some of the patients and hence we did not include volumetric and dosimetric data in this analysis.

## Results

### Patient inclusion

**Fig. 1:**
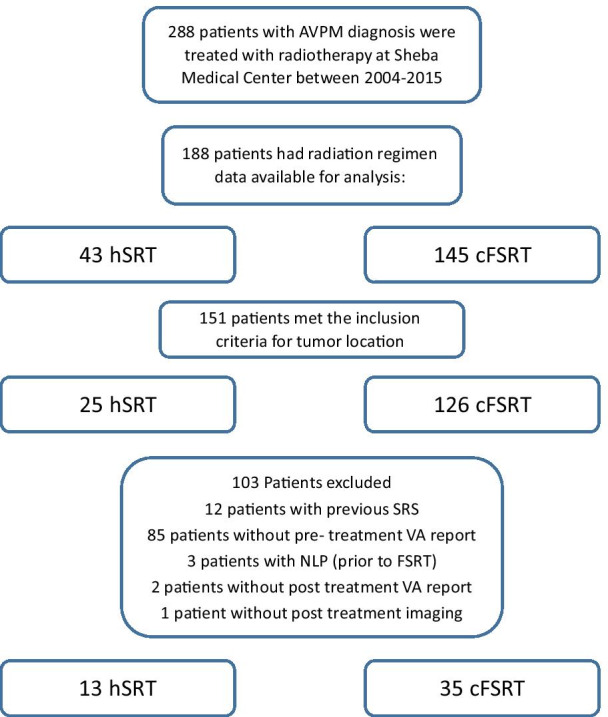
Inclusion and exclusion criteria of the study population. 288 patients with a radiological diagnosis of AVPM were treated with radiotherapy at Sheba Medical Center between 2004–2015. We included patients with a radiological diagnosis of meningioma whose tumors were in defined anatomical locations near the optic nerves and whose neuro-ophthalmology and neuroimaging data were available. We excluded patients having previous stereotactic radiosurgery treatment (SRS), lacking visual acuity documentation before treatment, having no light perception (NLP) prior to treatment, or undergoing additional treatment before the first neuro-ophthalmologic assessment. hSRT, hypofractionated stereotactic radiotherapy; cFSRT, conventionally fractionated stereotactic radiotherapy

Of 288 patients with a radiological diagnosis of AVPM treated with radiotherapy at Sheba Medical Center during 2004–2015, 48 patients were included in the final analysis: 13 in hSRT and 35 in cFSRT. See Fig. 1 for detailed exclusion criteria.

### Patient characteristics (Table 1)

There was no statistically significant difference between the groups in patients’ characteristics (Age, gender, previous surgery or radiotherapy to the same tumor, history of radiation therapy for tinea capitis or medical background associated with a risk for vision-related complications such as smoking, diabetes mellitus, hypertension or collagen vascular disorders). There was no statistically significant difference between the groups of patients showing evidence of optic nerve sheath involvement at baseline, 22 (63%) in cFSRT cohort and 6 (46%) in the hSRT cohort. Other locations of AVPM tumors involvement were equally distributed between the treatment groups and included planum sphenoidale, sphenoid wing, cavernous sinus, tuberculum sella and clinoid.

Median follow up from radiotherapy to last neuro-ophthalmologic evaluation was 55 months (range 18–162 months for all patients), with a significantly longer follow-up in the cFSRT cohort: median 73mo (range 21–162 months) in the cFSRT cohort vs. median 37mo (range 18–57 months) in the hSRT cohort (*p* < 0.0001). A neuro-ophthalmologist reviewed the medical records of all the patients known to have a systemic vision-threatening condition (e.g., diabetes mellitus, hypertension): No patient had evidence of ocular damage secondary to a systemic condition.

### Pre-treatment data

Neuro-ophthalmological evaluation prior to treatment revealed higher proportion of cFSRT patients with optic disc abnormality (atrophy/edema): 76% (25 of 33) compared with 27% (3 of 11) in the hSRT group (*p* = 0.009). The median pre-treatment better eye LogMAR was 0.10 in hSRT vs. 0.00 in cFSRT (*p* = 0.079). There was no statistically significant difference between cFSRT and hSRT regarding pretreatment worse eye LogMAR (*p* = 0.925), worse eye MD (*p* = 0.530), or better eye MD (*p* = 0.173). Cranial Nerve (CN) V1 involvement was found in 33% (3 of 9) in the hSRT group compared to 4% (1 of 28) in the cFSRT group (*p* = 0.038). Three patients in the cFSRT group were diagnosed with chronic ocular disease, compared with no patients in hSRT, with no significant difference between the groups: see Table 2.

**Table 2:** Neuro-ophthalmologic findings by treatment group

Neuro-ophthalmologic finding	Treatment group				*p*-value
	cFSRT		hSRT		
	n	Median (IQR) or n (%)	n	Median (IQR) or n (%)	
ΔLogMAR, worse eye	35	0.00 (− 0.05, 0.10)	13	0.05 (0.00, 0.50)	*0.092*
Clinically relevant VA deterioration (ΔLogMAR ≥ 0.2), worse eye	35	6 (17%)	13	6 (46%)	*0.061*
ΔMD, worse eye (dB)	16	2.4 (− 0.2, 4.5)	4	0.1 (− 4.2, 6.6)	0.682
ΔLogMAR, better eye	33	0.00 (− 0.02, 0.03)	12	0.00 (0.00, 0.07)	0.869
Clinically relevant VA deterioration (ΔLogMAR ≥ 0.2), better eye	33	1 (3%)	12	1 (8%)	0.467
ΔMD, better eye (dB)	21	0.6 (− 1.2, 1.7)	5	1.5 (− 4.7, 2.4)	> 0.999
*Optic disc abnormality (worse eye)*					
Pretreatment	33	Atrophy: 18 (55%) Edema: 7 (21%) Other: 0 (0%)	11	Atrophy: 3 (27%) Edema: 0 (0%) Other: 0 (0%)	*0.016*
Final evaluation	31	Atrophy: 20 (65%) Edema: 0 (0%) Other: 0 (0%)	11	Atrophy: 3 (27%) Edema: 0 (0%) Other: 0 (0%)	*0.043*
*Any optic disc abnormality (atrophy, edema, or other abnormality)*					
Pretreatment	33	25 (76%)	11	3 (27%)	*0.009*
CN V_1_ involvement					
Pretreatment	28	1 (4%)	9	3 (33%)	*0.038*
Final evaluation	29	5 (17%)	12	2 (17%)	> 0.999
Chronic ocular disease	33	3 (9%)	12	0 (0%)	0.553
Radiation-induced Optic Neuropathy (RION)					
During 1st year after treatment	24	1 (4%)	10	0 (0%)	> 0.999
Final evaluation	31	1 (3%)	11	0 (0%)	> 0.999
*Radiation retinopathy*					
During 1st year after treatment	25	1 (4%)	10	0 (0%)	> 0.999
Final evaluation	31	4 (13%)	11	1 (9%)	> 0.999

Values in italics denote findings of noteworthy differences between radiotherapy regiments

*AVPM* anterior visual pathway meningioma; *hSRT*, hypofractionated stereotactic radiotherapy; *cFSRT* conventionally fractionated stereotactic radiotherapy; *IQR* interquartile range; *LogMAR* logarithm of minimum angle of resolution; *MD* mean deviation; *∆LogMAR* final LogMAR minus pretreatment LogMAR; ΔMD = final MD minus pretreatment MD; *VA* visual acuity; *CN* cranial nerve

Histopathologic classification (WHO tumor grade) was documented in 13 cases with WHO Grade I or II tumors with a significant difference between the groups (Table 1). 13 patients were classified as having "unknown histology" (radiologic diagnosis without pathologic verification): Nine patients (56%) in the cFSRT, compared with four patients (40%) in the hSRT group.

### Radiotherapy schedule and dose

**Fig. 2:**
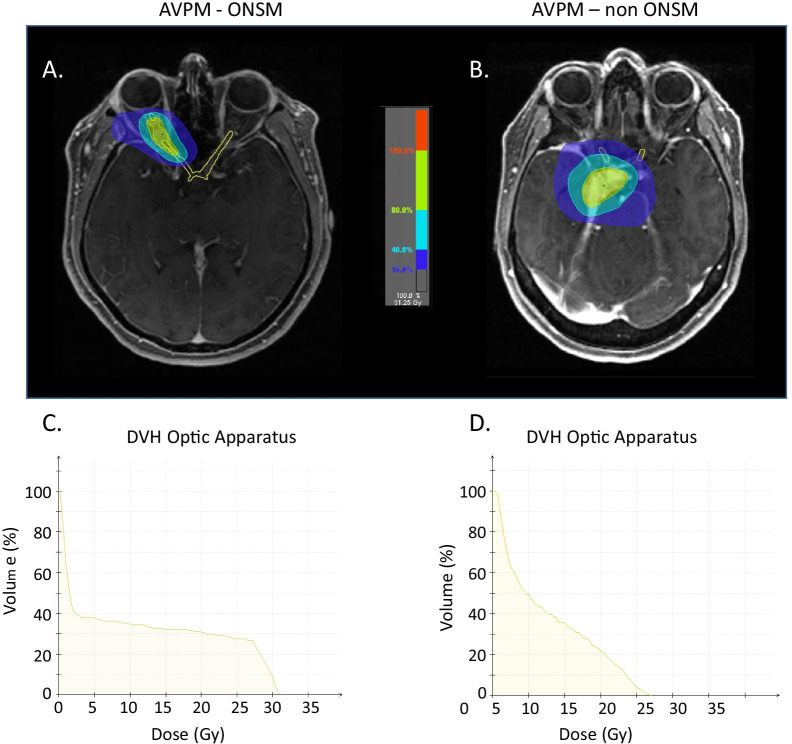
Radiation plan and Dose Volume Hisogram for AVPM patients with and without optic nerve sheath (ONSM) involvement. Both AVPM patients received hypofractionated radiosurgery (hSRT) in 5 sessions × 500 cGy, with maximum dose of 31.25 Gy. Optical apparatus (left and right optic nerve, optic chiasm) was marked in treatment plans of both patients and DVHs were calculated. **A** Radiation plan for Optic Nerve Sheath Meningioma (ONSM) patient. **B** Radiation plan for non-ONSM AVPM patient. Comparison of Optic apparatus DVH reveals that in the ONSM patient (**C**) a higher radiation dose was absorbed by the nerve sheath while in the perioptic non-ONSM AVPM patient (**D**) the same radiation dose to the tumor resulted in smaller dose to the nerve sheath. We believe the combination of pre-treatment nerve damage and the high dose to the nerve explain the increased visual damage we see in ONSM patients treated with hSRT and suggest this cohort should be treated with cFSRT

The number of radiotherapy fractions was five in the hSRT cohort, compared with 28 in almost all patients in the cFSRT cohort. The daily dose in the hSRT cohort was 500 centiGray (cGy) compared with 180 cGy in the cFSRT cohort (Fig. 2). Table 3 lists the radiation therapy characteristics.

**Table 3:** Characteristics of radiation therapy for AVPM by treatment group

Characteristic	Treatment group		*p*-value
	cFSRT (n = 35)	hSRT (n = 13)	
	28 × 180 cGy (80% IDL) EQD2 = 48–51.8 Gy Eq (α/β = 3)	5 × 450–600 cGy (80% IDL) EQD2 = 33.75–54 Gy Eq (α/β = 3)	
Treatment year	2010	2014	*p* < 0.001
Median (range)	(2004–2015)	(2013–2015)	
Time from pretreatment neuro-ophthalmologic evaluation to first day of radiation treatment (Median in months)	3	2	*p* = 0.487

*AVPM* anterior visual pathway meningioma; *cFSRT* conventionally fractionated stereotactic radiotherapy; *cGy* centigray; *hSRT* hypofractionated stereotactic radiotherapy; *IDL* Isodose Line; *EQD2* Two Gy bioequivalent radiation dose

### Neuro-ophthalmologic evaluation findings one year post-treatment

During the first year following treatment, 21 (66%) patients reported vision-related complaints or were found to have ocular complications, with no significant difference between the groups (*p* = 0.681). One patient in the cFSRT cohort developed retinal bleeding in the involved eye; we considered this case as radiation retinopathy, although it is unclear whether diabetic changes had a role in this complication. RION was diagnosed in one cFSRT patient (2% of all patients in the study) and no patients in the hSRT cohort (*p* > 0.999). This patient's worse eye VA remained stable during long-term follow-up (ΔLogMAR = 0.08), but the VF deteriorated (ΔMD =  − 9.4).

### Last Neuro-ophthalmologic evaluation findings

No difference was found between treatment regimens in the last assessment worse eye LogMAR (*p* = 0.327), worse eye MD (*p* = 0.935), or better eye LogMAR (*p* = 0.115). The median of the last assessment better eye MD was − 1.6 (IQR: − 4.4, − 0.3. n = 26) in cFSRT, compared with − 4.7 (IQR: − 11.3, − 1.6. n = 8) in hSRT (*p* = 0.043), reflecting better VF results in cFSRT (but see later comments on missing MD data). The proportion of patients with involved side optic disc abnormality in the cFSRT cohort was higher (65%) than in the hSRT cohort (27%) (*p* = 0.043), similar to the proportion before irradiation (Table 2).

Five cases (12%) were diagnosed with radiation retinopathy: 4 patients (13%) in the cFSRT cohort vs. 1 patient (9%) in the hSRT cohort (*p* > 0.999).

### Change in visual acuity and visual field between pre-treatment and final evaluation

The median change in VA (ΔLogMAR) in the worse eye during the last neuro-ophthalmologic assessment compared to the pre-irradiation evaluation was 0.00 (IQR − 0.05, 0.10) in the cFSRT group and 0.05 (IQR 0.00, 0.50) in the hSRT group, suggesting a better long term outcome in the cFSRT cohort (*p* = 0.092). Of the 48 patients in the study, 12 (25%) had a clinically relevant deterioration in VA (ΔLogMAR ≥ 0.2) in the involved eye in the last neuro-ophthalmologic assessment: six patients (17%) in the cFSRT cohort and six patients (46%) in the hSRT cohort (*p* = 0.061) (Table 2).

An evaluation of the changes in visual functions—between baseline and the last assessment—is summarized in Table 4. In the hSRT cohort, the median LogMAR of worse eyes in the final assessment was 0.30, compared with 0.10 at baseline, suggesting post-treatment VA deterioration after hSRT. However, this difference did not reach statistical significance (Wilcoxon Signed-Ranks test, *p* = 0.068). In the cFSRT cohort, the scores of the MD values of the worse eye in the last assessment were significantly better than the scores of MD values of the worse eye at baseline (Wilcoxon Signed-Ranks test, *p* = 0.034). However, this result is based on only 16 cases (of 35), and it may be biased due to the remaining cases' missing data.

**Table 4:** Change in visual functions between pretreatment evaluation and final evaluation in AVPM

Radiotherapy regimen		Final LogMAR (worse eye) minus pretreatment LogMAR (worse eye)	Final MD (worse eye) minus pretreatment MD (worse eye)	Final LogMAR (better eye) minus pretreatment LogMAR (better eye)	Final MD (better eye) minus pretreatment MD (better eye)
cFSRT	*p*-value*	0.726	0.034	0.740	0.434
		(n = 35)	(n = 16)	(n = 33)	(n = 21)
hSRT	*p*-value*	0.068	0.715	0.893	0.686
		(n = 13)	(n = 4)	(n = 12)	(n = 5)

*cFSRT* conventionally fractionated stereotactic radiotherapy; *hSRT* hypofractionated stereotactic radiotherapy; *LogMAR* logarithm of minimum angle of resolution; *MD* mean deviation

^*^Wilcoxon's signed-rank test, two-tailed

VA deterioration was more evident after hSRT than after cFSRT, although findings did not reach statistical significance. In order to explore a possible contribution of tumor location we re-analyzed the data for visual function variables in a subset of patients (hSRT, n = 11, cFSRT, n = 19) in which *no optic nerve sheath involvement* was documented in the pre-treatment imaging test. No patient in this subgroup developed RION or radiation retinopathy. In this subgroup analysis no statistically significant difference was found between hSRT and cFSRT in the proportion of complications or complaints related to vision in the first year after radiation therapy (*p* = 0.628), change in worse eye VA (∆LogMAR, *p* = 0.471), change in worse eye VF(∆ MD, *p* = 0.714), the proportion of patients with a clinically relevant deterioration (ΔLogMAR ≥ 0.2) in the involved eye VA (*p* = 0.417), or in the proportion of patients with PD in imaging (*p* > 0.999).

### Imaging findings

Evidence of disease progression (PD) in imaging during the follow-up period was found in 5 patients (14%) in the cFSRT cohort, compared with 2 (15%) in the hSRT cohort (*p* > 0.999). Since the median duration from the last MRI scan to the last neuro-ophthalmologic assessment for all patients was two months, we believe that the imaging reports accurately reflect the tumor's state at the time of this evaluation.

## Discussion

We studied patients treated with radiotherapy with a threat to visual function due to tumor location (in or near the AVP). The results are intended to support therapeutic decision-making in patients with AVPM not amenable to surgical treatment by pointing to a radiation therapy regimen associated with better post-treatment vision functions, to improve, even if only slightly, the prognosis of their vision. We examined the association between radiation regimen and visual function results and radiological PD, in patients with AVPM. Patients in the cFSRT group were more likely to have preserved VA after radiotherapy compared to hSRT group, although they had more optic disc abnormalities before and after the treatment (Table 2).

The proportion of patients (25%) who had a clinically relevant VA deterioration was higher than we expected from previous publications on cFSRT (see Additional File 1) and hSRT [4, 6, 23, 26–29]. This may be due to differences in measured and reported variables (mainly VA), follow-up length, prognostic factors such as histology, equipment, and radiotherapy treatment plans.

While there was no difference in pre-treatment visual function between patients assigned to each radiotherapy regimen, VA deterioration was more evident in hSRT than in cFSRT, although findings did not reach statistical significance. This difference disappeared when we examined a subset of patients without optic nerve sheath (ONSM) involvement, suggesting a possible relationship between the treatment regimen, tumor location, and effects on visual functions. We propose the use of hSRT in patients with ONSM involvement may be associated with a worse visual outcome, though our analysis relies on a small number of patients and should be examined in further studies, that will separate ONSM from other AVPM tumors.

RION rate in the first year after radiation therapy is consistent with the literature [16, 30]. This impairment manifested in VF deterioration without an accompanying clinically relevant VA deterioration, demonstrating the importance of VF evaluation in the follow-up of patients with AVPM treated with radiotherapy. Most radiation retinopathy cases were not diagnosed in the first year post-treatment, consistent with previous publications about the delayed onset and slow progression of this condition [13].

Of the 48 cases in the study, WHO grade was documented in 13 cases, mostly WHO grade I. We assume that the vast majority of other tumors was also WHO Grade I meningioma. In this cohort we find that both radiation regimens were efficient in controlling AVPM in imaging, as 85% of patients were without PD at the last assessment, with no significant difference between hSRT and cFSRT at a median follow-up period of 4.5 years after treatment. This result agrees with previous publications stating that five to ten years after radiation therapy, a local control rate of 68% to 100% was reached in WHO Grade I meningioma (presumed or histologically verified) [11, 14, 31].

Conti et al. [7] reviewed previous publications reporting hSRT treatment of AVPM and described "controlled tumor growth" and lack of "optic nerve toxicity" in a series of 25 patients treated with 2–5 doses of 4-10 Gy each. Hiniker et al. [18] summarized previous publications (including Emami et al. [19] and later information from QUANTEC [20]) and reported results of treating peri-optic tumors by hSRT in up to five fractions; they suggested both hSRT and SRS were safe treatment options. Marchetti et al. [21] reported the results of treating 143 patients with hSRT (25 Gy in 5 Gy sessions over five consecutive days). VA change (worsening or improvement) was defined as one or more Snellen lines. VF deterioration was defined as an increase in the defect area. The authors reported a visual worsening rate of 7.4% (5.1% after excluding cases with PD). Conti et al. [11] reported a multicenter retrospective heterogeneous cohort of 341 patients with skull-base meningiomas, and compared cFSRT to hSRT. Visual toxicity was reported for one case (0.49%) of mild visual disturbance in hSRT vs. one case (0.7%) of moderate optical pathway toxicity in cFSRT. Most recently, Marchetti et al. [31] published results of 167 patients treated with 5X5Gy hSRT. The authors reported an overall visual worsening rate of 5.5%, or 3.7% if excluding PD patients with no details regarding the test used.

Differences in the length of follow up, measures of toxicity and separation of ONSM from other AVPM tumors make it complicated to draw unequivocal conclusions from the published literature. We chose dose regimen using EQD2 calculations (Table 3) which did not exceed the allowed dose to optic structures with the hypo-fractionated schedules.

We initiated this retrospective analysis due to concerns from our Neuro-Ophthalmology colleagues, regarding the safety of hypo-fractionated schedules especially with optic nerve sheath involvement.

This study suffers several weaknesses due to its retrospective nature, including missing data for many patients, and the differences between study cohorts in treatment year and follow-up length. These may confound the results related to visual function and PD. However, unlike previous reports, our study has the strengths of using neuro-ophthalmologists' meticulous assessment of visual function and quantitative deterioration criterion, which allowed us to identify cases that may not have been identified if data relied solely on patient reporting. Other strengths include evaluating prognostic factors such as pathology reports that are not available in many studies, having long-term follow-up of the patients, and employing a pre- and post-treatment analytic design.

## Conclusions

Although this study did not confirm a statistically significant association between the radiation therapy regimen (hSRT vs. cFSRT) and visual function outcomes in post-treatment years, our findings using a comprehensive and meticulous investigation of visual outcomes suggest that cFSRT may be associated with less VA toxicity. Given the small sample and retrospective nature of our study, caution is needed in concluding which is the best regimen to be used. Based on our results, we chose to keep the traditional cFSRT regimen for ONSM and use hSRT only for tumors adjacent to the AVPM. We suggest this group of patients requires a multidisciplinary follow-up with meticulous pre- and post-treatment neuro-ophthalmologic evaluation, along with magnetic resonance imaging and patient-reported outcomes. Such information, along with well-designed prospective studies, may improve our understanding of the relationship between radiation regimen and long-term outcomes in AVPM. We believe our results indicate the importance of using thorough clinical investigation when adopting new radiation regimens.

## Supplementary Information

**Additional file 1: Supplementary Table 1**: Patients suffering from permanent deterioration in vision as reported in published studies of cFSRT treatment for AVPM. A table summarizing 17 studies describing cFSRT treatment for AVPM, focusing on findings of deterioration in patients. The file includes also the list of references.

## Data Availability

The datasets analyzed during the current study are available from the corresponding author on reasonable request.

## Abbreviations

AVP: Anterior Visual Pathway
AVPM: Anterior Visual Pathway Meningioma
cFSRT: Conventional Fractionated Stereotactic Radiotherapy
cGy: Centi-Gray
FSRT: Fractionated Stereotactic Radiotherapy
Gy: Gray
hSRT: Hypofractionated Stereotactic Radiotherapy
IQR: Interquartile range
LogMAR: Logarithm of the Minimum Angle of Resolution
MD: Mean deviation
MRI: Magnetic resonance imaging
NLP: No light perception
ONSM: Optic nerve sheath meningioma
PD: Disease progression
RION: Radiation-induced optic neuropathy
VA: Visual acuity
VF: Visual field
WHO: World Health Organization
