# Supplementary material for: Neuro-ophthalmologic outcomes of standard versus hypo-fractionated stereotactic radiotherapy of AVPM
Source: Radiat Oncol. 2021 Aug 28;16:166. doi: 10.1186/s13014-021-01879-2 (PMC8403384; doi:10.1186/s13014-021-01879-2)
Supplement: Supplementary file 1 — Additional file 1: Supplementary Table 1: Patients suffering from permanent deterioration in vision as reported in published studies of cFSRT treatment for AVPM. A table summarizing 17 studies describing cFSRT treatment for AVPM, focusing on findings of deterioration in patients. The file includes also the list of references. [file 13014_2021_1879_MOESM1_ESM.docx]

**Supplementary Table 1 – Patients suffering from permanent deterioration in vision as reported in published studies of cFSRT treatment for AVPM**

| Comments | Patients with permanent deterioration in vision (n) | Patients(n) | Year | Study |
| --- | --- | --- | --- | --- |
| One patient developed transient optic neuropathy, which responded to glucocorticoid treatment. | 0 | 16 | 2012 | Stiebel-Kalish et al.^1^ |
| One patient with optic nerve involvement suffered deterioration of vision 18mo after treatment. | 1 | 41 | 2002 | Jalali et al.^2^ |
| One patient suffered deterioration of vision 4mo after IMRT treatment. | 1 | 20 | 2003 | Pirzkall et al.^3^ |
| Seven patients suffered from reduced vision, and four patients suffered a loss of visual field. In all cases, deterioration was in symptoms that existed before treatment. | 11* | 317 | 2005 | Milker-Zabel et al.^4^ |
| Three patients reported deterioration of vision without ophthalmological findings. | 0 | 57 | 2006 | Milker-Zabel et al.^5^ |
|  | 0 | 183† | 2006 | Henzel et al.^6^ |
| Treatment-induced loss of vision was seen in one re-irradiated patient with a Grade 3 meningioma 9mo after IMRT treatment. | 1 | 94 | 2007 | Milker-Zabel et al.^7^ |
| One patient suffered progressive concentric decreasing of the visual field on the side of the irradiated ONSM. | 1 | 32 | 2009 | Milker-Zabel et al.^8^ |
|  | 0 | 5 | 2002 | Liu et al.^9^ |
|  | 0 | 100 | 2009 | Litre et al.^10^ |
| One patient suffered a loss of visual acuity. | 1 | 30 | 2006 | Brell et al.^11^ |
|  | 0 | 45 | 2004 | Selch et al.^12^ |
| One patient developed persistent third nerve paresis. | 0 | 53 | 2010 | Metellus et al.^13^ |
| One patient developed radiation retinopathy. | 1 | 9 | 2011 | Metellus et al.^14^ |
| One patient with an initial improvement in visual acuity later suffered deterioration. Another patient suffered deterioration in visual acuity that remained stable for four years. | 2 | 23 | 2003 | Narayan et al.^15^ |
| Two patients developed blindness, and one patient developed optic neuritis post-treatment. | 3 | 30 | 2002 | Andrews et al.^16^ |
| One patient developed moderate optical pathway toxicity | 1 | 136 | 2019 | Conti et al.^17^ |

cFSRT, conventionally fractionated stereotactic radiotherapy; AVPM, anterior visual pathway meningioma; ONSM, optic nerve sheath meningioma; IMRT, intensity-modulated radiotherapy. * The number of patients might be smaller due to possible overlap between reports of "reduced vision" and "loss of visual fields." †183 out of 224 patients were treated with conventional FSR.

References for Supplementary Table 1

1. Stiebel-Kalish H, Reich E, Gal L, et al. Visual outcome in meningiomas around anterior visual pathways treated with linear accelerator fractionated stereotactic radiotherapy. *Int J Radiat Oncol Biol Phys*. 2012;82(2):779-788. doi:10.1016/j.ijrobp.2010.12.017

2. Jalali R, Loughrey C, Baumert B, et al. High precision focused irradiation in the form of fractionated stereotactic conformal radiotherapy (SCRT) for benign meningiomas predominantly in the skull base location. *Clin Oncol (R Coll Radiol)*. 2002;14(2):103-109. doi:10.1053/clon.2001.0040

3. Pirzkall A, Debus J, Haering P, et al. Intensity modulated radiotherapy (IMRT) for recurrent, residual, or untreated skull-base meningiomas: preliminary clinical experience. *Int J Radiat Oncol Biol Phys*. 2003;55(2):362-372. doi:10.1016/s0360-3016(02)03809-9

4. Milker-Zabel S, Zabel A, Schulz-Ertner D, Schlegel W, Wannenmacher M, Debus J. Fractionated stereotactic radiotherapy in patients with benign or atypical intracranial meningioma: long-term experience and prognostic factors. *Int J Radiat Oncol Biol Phys*. 2005;61(3):809-816. doi:10.1016/j.ijrobp.2004.07.669

5. Milker-Zabel S, Zabel-du Bois A, Huber P, Schlegel W, Debus J. Fractionated stereotactic radiation therapy in the management of benign cavernous sinus meningiomas : long-term experience and review of the literature. *Strahlenther Onkol*. 2006;182(11):635-640. doi:10.1007/s00066-006-1548-2

6. Henzel M, Gross MW, Hamm K, et al. Stereotactic radiotherapy of meningiomas: symptomatology, acute and late toxicity. *Strahlenther Onkol*. 2006;182(7):382-388. doi:10.1007/s00066-006-1535-7

7. Milker-Zabel S, Zabel-du Bois A, Huber P, Schlegel W, Debus J. Intensity-modulated radiotherapy for complex-shaped meningioma of the skull base: long-term experience of a single institution. *Int J Radiat Oncol Biol Phys*. 2007;68(3):858-863. doi:10.1016/j.ijrobp.2006.12.073

8. Milker-Zabel S, Huber P, Schlegel W, Debus J, Zabel-Du Bois A. Fractionated stereotactic radiation therapy in the management of primary optic nerve sheath meningiomas. *J Neurooncol*. 2009;94(3):419-424. doi:10.1007/s11060-009-9874-8

9. Liu JK, Forman S, Hershewe GL, Moorthy CR, Benzil DL. Optic nerve sheath meningiomas: visual improvement after stereotactic radiotherapy. *Neurosurgery*. 2002;50(5):950-957. doi:10.1097/00006123-200205000-00006

10. Litré CF, Colin P, Noudel R, et al. Fractionated stereotactic radiotherapy treatment of cavernous sinus meningiomas: a study of 100 cases. *Int J Radiat Oncol Biol Phys*. 2009;74(4):1012-1017. doi:10.1016/j.ijrobp.2008.09.012

11. Brell M, Villà S, Teixidor P, et al. Fractionated stereotactic radiotherapy in the treatment of exclusive cavernous sinus meningioma: functional outcome, local control, and tolerance. *Surg Neurol*. 2006;65(1):24-28. doi:10.1016/j.surneu.2005.06.027

12. Selch MT, Ahn E, Laskari A, et al. Stereotactic radiotherapy for treatment of cavernous sinus meningiomas. *Int J Radiat Oncol Biol Phys*. 2004;59(1):101-111. doi:10.1016/j.ijrobp.2003.09.003

13. Metellus P, Batra S, Karkar S, et al. Fractionated conformal radiotherapy in the management of cavernous sinus meningiomas: long-term functional outcome and tumor control at a single institution. *Int J Radiat Oncol Biol Phys*. 2010;78(3):836-843. doi:10.1016/j.ijrobp.2009.08.006

14. Metellus P, Kapoor S, Kharkar S, et al. Fractionated conformal radiotherapy for management of optic nerve sheath meningiomas: long-term outcomes of tumor control and visual function at a single institution. *Int J Radiat Oncol Biol Phys*. 2011;80(1):185-192. doi:10.1016/j.ijrobp.2010.01.034

15. Narayan S, Cornblath WT, Sandler HM, Elner V, Hayman JA. Preliminary visual outcomes after three-dimensional conformal radiation therapy for optic nerve sheath meningioma. *Int J Radiat Oncol Biol Phys*. 2003;56(2):537-543. doi:10.1016/s0360-3016(03)00005-1

16. Andrews DW, Faroozan R, Yang BP, et al. Fractionated stereotactic radiotherapy for the treatment of optic nerve sheath meningiomas: preliminary observations of 33 optic nerves in 30 patients with historical comparison to observation with or without prior surgery. *Neurosurgery*. 2002;51(4):890-894. doi:10.1097/00006123-200210000-00007

17. Alfredo C, Carolin S, Güliz A, et al. Normofractionated stereotactic radiotherapy versus CyberKnife-based hypofractionation in skull base meningioma: a German and Italian pooled cohort analysis. *Radiat Oncol*. 2019;14(1):1-9. doi:10.1186/s13014-019-1397-7
